# Non-Native Flora of the Mediterranean Lesvos Island (East Aegean, Greece): Floristic Analysis, Traits, and Assessment

**DOI:** 10.3390/plants13233375

**Published:** 2024-11-30

**Authors:** Alexandros Galanidis, Ioannis Bazos, Panayiotis G. Dimitrakopoulos

**Affiliations:** 1 Biodiversity Conservation Laboratory, Department of Environment, University of the Aegean, 81100 Mytilene, Greece; pdimi@env.aegean.gr; 2 Department of Ecology and Systematics, Faculty of Biology, School of Sciences, National and Kapodistrian University of Athens, 15784 Athens, Greece; ibazos@biol.uoa.gr

**Keywords:** alien species, invasive species, survey, checklists, islands, life-history traits, origin, pathways, species–area curves

## Abstract

A checklist of Lesvos Island’s non-native vascular flora is presented. Through the literature and a roadside survey, we recorded 187 non-native plant taxa, representing 12% of the total regional flora. A total of 37 taxa were reported for the first time for Lesvos, including three taxa that are also new to the Greek non-native flora. The dominant families were Fabaceae, Asteraceae, Poaceae, Amaranthaceae, and Solanaceae. *Amaranthus*, *Oxalis*, *Solanum*, *Opuntia*, and *Prunus* were the most species rich genera. Most taxa were neophytes, naturalized and perennial, while phanerophytes and therophytes were the predominant life forms. Animal (insect) pollination was the principal pollination mode, whereas the main dispersal mechanism was zoochory, with most taxa forming a capsule. The majority of the taxa preferred agricultural and ruderal habitats and originated from the Americas and Asia. The primary introduction pathway was escape from confinement, concerning taxa introduced for ornamental and agricultural purposes.

## 1. Introduction

Biological invasions are an expanding component of global environmental change [1]. They can cause serious ecological, socio-economic, and health problems and affect an ecosystem’s functioning, structure, composition, and biodiversity [2]. Driven by the globalization of trade and transport [3,4], land use changes [5,6], climate alteration [7], and access to new source pools [8], non-native species are increasing in number, population size, and spread. Future projections predict an increase in alien species numbers worldwide, especially in Europe [9].

In contrast to other Mediterranean-type ecosystems of the world, the Mediterranean Basin is regarded as less susceptible to invasion due to its long and gradual history of human intervention [10] and the fact that more European species were intentionally introduced by European settlers to the colonies than the opposite [11]. Moreover, compared to temperate ecosystems, the summer drought stress limitations in the area appear to prevent the establishment of non-adapted plants, a remark that is consistent with recent studies indicating lower numbers of alien species in the Mediterranean than in temperate Europe [12].

Islands are defined by distinct boundaries, where most biotic assemblages and ecological processes in them are fundamentally restricted by obvious geographical constraints [13]. Excluding the tropics, the Mediterranean Basin is the richest area in islands than anywhere else, with more than 10,000 islands and islets. Mediterranean islands are considered biodiversity hotspots that are rich in rare habitats and endemic and range-restricted plant species [11,14]. Compared to continental Europe, Mediterranean islands have been considered less invaded, as revealed by crosschecks between their floras [12]. However, Mediterranean islands tend to be more vulnerable to impacts by alien taxa than other comparable in size mainland areas [11,15]. Biogeographical (e.g., unsaturated island communities, smaller regional species pool, lower habitat diversity), ecological (e.g., less competitive native species, lack of key plant functional groups), and socio-economic factors (e.g., abandonment of agricultural land, development of tourism, intense anthropogenic activity particularly in coastal and riparian habitats) contribute to this pattern [11,12,15,16,17].

Furthermore, recent studies showed a drastic rise in the number of non-native species in both Europe and the Mediterranean Basin [3,18]. This trend has been considerably accelerated for Mediterranean islands regarding the establishment success and distribution range of their alien species [15]. Although the levels of invasion in Mediterranean insular ecosystems are considered context-dependent [17] and habitat-specific [19], various factors linked to human presence are also known to facilitate alien species introduction and establishment in the area. Human population density [16,20], road networks [21,22,23], transport infrastructures [16,23], tourism development [14,21,23], or the percentage of agricultural areas and artificial surfaces [23] are eventually connected to increased propagule pressure that is considered to promote the colonization and persistence of alien species [24].

Being geographically isolated, islands miss opportunities to be naturally colonized by non-native species, and this fact reveals, in greater strength, the role of humans as a key dispersal vector. The deliberate use of alien species in agriculture, forestry, horticulture, or even as ornamental plants has been intensified in the Mediterranean since the middle of the 20th century [11]. Along with various unintentional pathways, such as transport contaminants on seed supplies or transport stowaways on vehicles or machinery, Mediterranean islands are facing increased introduction pressure, making their ecosystems some of the most threatened by biological invasions globally.

Checklists are an essential management tool for the protection and conservation of biodiversity and ecosystem functions, constituting a structural part of species richness and population trend monitoring systems. Such lists link taxonomic, spatial, and temporal information and provide a benchmark for detecting the most vulnerable areas and species, support decision-making, and ensure the efficient allocation of resources aimed at avoiding or mitigating the negative effects of biological invasions [25,26]. In this work, along with the compilation of the non-native flora checklist for Lesvos, one of the largest Mediterranean islands, we aim to analyze various life-history, reproductive, and dispersal traits, habitat preferences, chorological data, and introduction pathways. Furthermore, we explore possible correlations or dependencies of these parameters with the current position of non-native taxa on the introduction–naturalization–invasion continuum (INIC).

## 2. Results

### 2.1. Species Richness and Taxonomy

A total of 187 taxa (species and subspecies) were recorded. Most of them (118; 63%) were observed in the field, while 69 taxa records (37%) were obtained from the literature (Table S1). The alien plant species richness of Lesvos Island corresponds to 11.6% of the total flora on the island; when excluding casual aliens, this percentage falls to 7.5%. Among the recorded taxa, there were 37 new entries for Lesvos Island (e.g., with no records previously reported), including three records that were new for Greece as well (Table S1). The majority of these new taxa were perennial, woody, casual neophytes.

**Figure 1:**
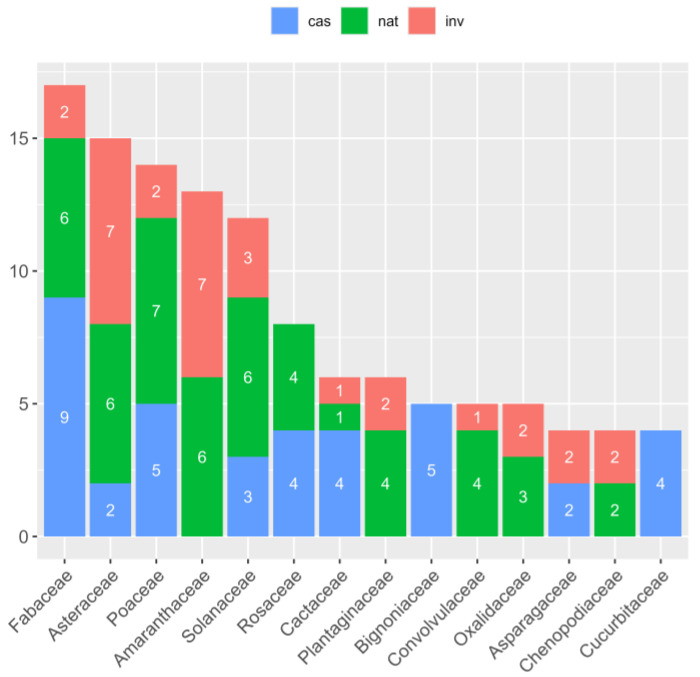
Number of non-native taxa per family across invasion status categories. cas: casual; nat: naturalized; inv: invasive taxa.

The taxa belong to 60 different families with the most abundant ones being Fabaceae (17 taxa; 9.1%), Asteraceae (15 taxa; 8%), Poaceae (14 taxa; 7.5%), Amaranthaceae (13 taxa; 7%), Solanaceae (12 taxa; 6.4%), Rosaceae (8 taxa; 4.3%), and Cactaceae and Plantaginaceae (6 taxa; 3.2% each). The top five families were also the most abundant in naturalized taxa, while the highest number of invasive taxa were found for Amaranthaceae (seven taxa; 54% of the family’s taxa) and Asteraceae (seven taxa; 46.7% of the family’s taxa) (Figure 1). Thirty families (50%) were represented by one taxon only.

The total number of different genera was 137, and the families with the highest numbers of genera were Fabaceae (15 genera), Poaceae (13 genera), Asteraceae (9 genera), Solanaceae (6 genera), and Bignoniaceae and Rosaceae (5 genera each). Thirty-three families (55%) were represented by one genus only. The most abundant genus was *Amaranthus*, with 13 taxa, followed by *Oxalis* and *Solanum* (five taxa each) and *Opuntia* and *Prunus* (four taxa each). About 60% of the genera (112 taxa) were represented by one taxon only.

### 2.2. Residence and Invasion Status

#### 2.2.1. Residence Status

**Figure 2:**
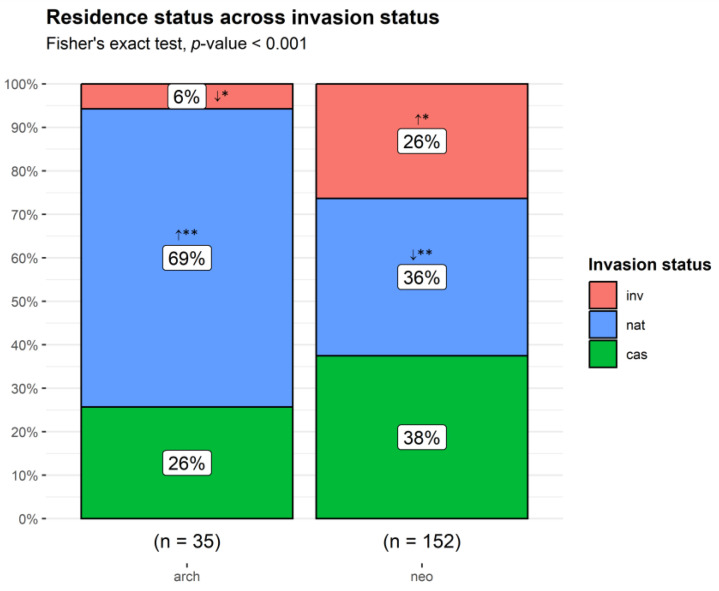
Proportion of non-native taxa by residence status across invasion status categories. arch: archaeophyte; neo: neophyte. cas: casual; nat: naturalized; inv: invasive taxa. **↑** observed counts higher than expected by chance; **↓** observed counts lower than expected by chance. ** *p* < 0.01; * *p* < 0.05.

Most of the taxa were neophytes (152; 81%). Archaeophytes were mainly naturalized (69%), while neophytes were more equally distributed across INIC (38% casuals, 36% naturalized, and 26% invasive). Established taxa (naturalized and invasive) accounted for 74% of the archaeophytes and 63% of the neophytes. There were significant differences in the counts of taxa per residence status between invasion status categories (Fisher’s exact test, *p* < 0.001). Invasives were over-represented among neophytes and under-represented among archaeophytes, while for naturalized taxa, the outcome was reversed, as more taxa than expected were observed for archaeophytes and fewer for neophytes (Figure 2).

#### 2.2.2. Invasion Status

Of the 187 taxa, 35% were casual, 42% naturalized, and 23% invasives, resulting in a 65% majority of the taxa being already established on the island. The families with the highest number of invasive taxa were Amaranthaceae and Asteraceae (seven taxa each), while most of the naturalized taxa were found in Poaceae (seven taxa each) and Amaranthaceae, Asteraceae, Fabaceae, and Solanaceae (six taxa each).

Comparing the top five richest families between the whole non-native flora and the invasion status categories (established taxa included), similar patterns were found, apart from the invasive taxa where Aizoaceae and Euphorbiaceae, although low in the whole alien flora ranking, emerged in the top five families (with three taxa each).

### 2.3. Life-History Traits

#### 2.3.1. Life Span

Most of Lesvos’ alien plants were perennials (64%), while 36% were annual, and one taxon was biennial. Most perennial and annual alien taxa were naturalized (42% and 43%, respectively).

#### 2.3.2. Growth Form

**Figure 3:**
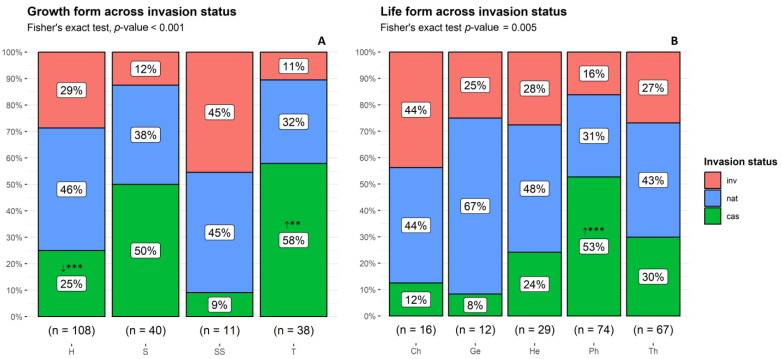
Proportion of non-native taxa by (**A**) growth form and (**B**) life form across invasion status categories. H: herb; S: shrub; SS: subshrub; T: tree. Ch: chamaephyte; Ge: geophyte; He: hemicryptophyte; Ph: phanerophyte; Th: therophyte. cas: casual; nat: naturalized; inv: invasive taxa. **↑:** observed counts higher than expected by chance; **↓** observed counts lower than expected by chance. *** *p* < 0.001; ** *p* < 0.01.

The most common growth form was herbaceous (58%), followed by shrub (21%), tree (20%), and subshrub (6%). There were significant differences in the counts of taxa per growth form between the invasion status categories (Fisher’s exact test, *p* < 0.001). These differences were due to herbs, where the observed counts were lower than expected by chance for casual species, and due to trees, where casuals were more than expected (Figure 3A).

#### 2.3.3. Life Form

In total, phanerophytes (40%) and therophytes (36%) were the most frequent life forms, followed by hemicryptophytes (16%), chamaephytes (9%), and geophytes (6%). Woody plants (phanerophytes and chamaephytes) represented 48% of the taxa. Among the established taxa, therophytes were dominant (39%), followed by phanerophytes (29%) and hemicryptophytes (19%). There were significant differences in the counts of taxa per life form between the invasion status categories (Fisher’s exact test, *p* = 0.005). These differences were due to phanerophytes, where the observed counts for casual species were higher than expected by chance (Figure 3B).

#### 2.3.4. Dispersal Mechanisms

The main dispersal mechanism for Lesvos aliens was zoochory (49%), followed by barochory (25%) and anemochory (24%), with no significant differences among the invasion status groups. Zoochory was also the main dispersal mechanism for invasive (52%) and naturalized (47%) taxa. The majority of the taxa (89%) were dispersed by a single mechanism.

#### 2.3.5. Pollination

Most of the taxa (57%) relied on animal pollinators, predominantly insects (105 taxa). Self-pollination was followed by 42% of the taxa, while wind pollination occurred in 12% of the taxa. Although there were no significant differences between categories, more casuals than naturalized or invasives and more archaeophytes than neophytes were pollinated by animals.

#### 2.3.6. Flowering

**Figure 4:**
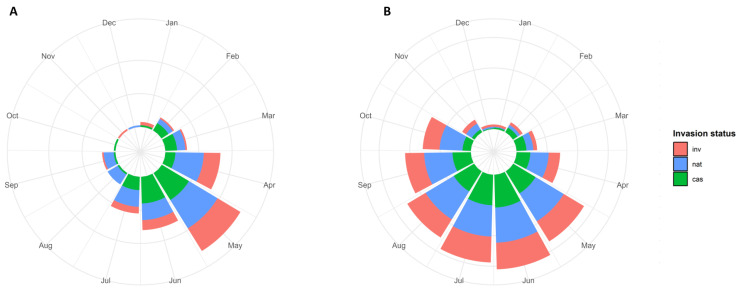
Circular histograms for (**A**) the start of flowering time and (**B**) the number of taxa flowering per month across invasion categories. Each triangle represents the number of non-native taxa that flower in that month. cas: casual; nat: naturalized; inv: invasive taxa.

The taxa mainly started to flower in May (29%), followed by April (18%), June (17%), and July (12%), whereas fewer taxa were found to enter their flowering phase in October, November, and December (one, one, and two taxa, respectively) (Figure 4A). The flowering response data showed that most of the taxa (67%) flowered in June, followed by July (61%), May (58%), and August (55%). The lowest response was observed in December and January (3% each) (Figure 4B). For nearly half of the taxa (46%), the flowering span lasted two to three months.

#### 2.3.7. Fruit Types

Capsule was the predominant fruit type (35%), followed by taxa that produced berries (17%), achenes (16%), and legumes (10%). Most of the invasive (45%) and naturalized (35%) taxa likewise produced capsules as their fruit type. Significant differences for fruit type were found between the invasion status categories (Fisher’s exact test, *p* = 0.0015), as fewer casual taxa than expected by chance were found forming an achene.

### 2.4. Habitats

**Figure 5:**
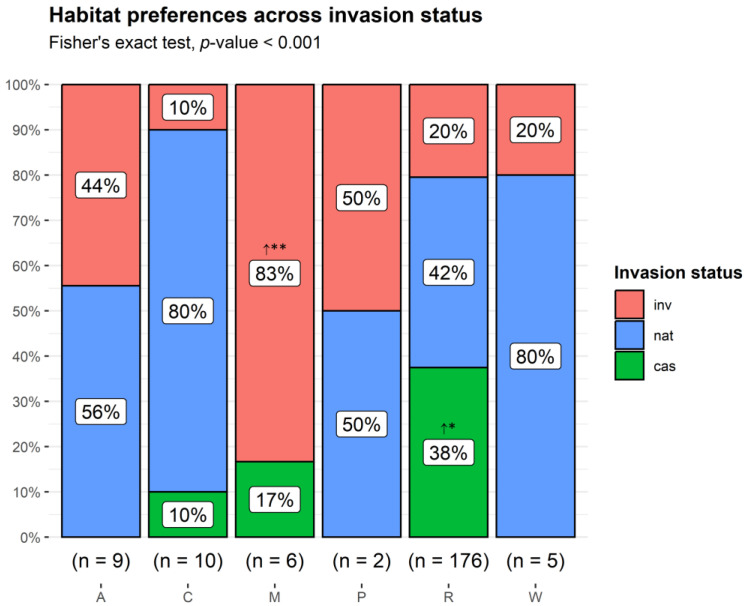
Proportion of non-native taxa by habitat type preferences across invasion status categories. A: freshwater; C: cliffs, rocks, walls, ravines, boulders; M: coastal; P: xeric Mediterranean phrygana and grasslands; R: ruderal and agricultural; W: woodlands and scrub. cas: casual; nat: naturalized; inv: invasive taxa. **↑** observed counts higher than expected by chance. ** *p* < 0.01; * *p* < 0.05.

Nearly all of the alien plants recorded on Lesvos Island showed a preference for ruderal and agricultural habitats (94%), with the remaining taxa found in rather low proportions to other habitats; 10 taxa were found in rocky, 9 taxa in freshwater, 6 taxa in coastal, 5 taxa in woodland, and 2 taxa in phryganic habitats. Established taxa were found in all habitats. There were significant differences in the counts of taxa per habitat between the invasion status categories (Fischer exact test *p* < 0.001). More casuals than expected by chance were found in ruderal and agricultural habitats, while invasive taxa were over-represented in coastal habitats (Figure 5).

### 2.5. Origin

**Figure 6:**
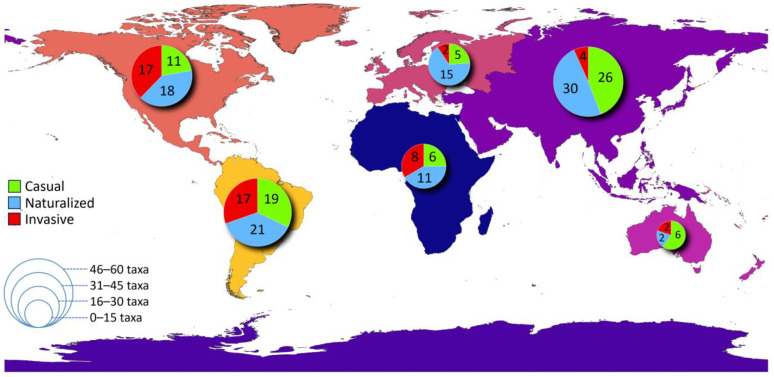
Global map with the continent of origin for non-native plant taxa on Lesvos Island. The size of the pie indicates the number of taxa originating from each continent. Pie sector size indicates the number of taxa per invasion status category. Map created with QGIS 3.34 “Prizren”.

**Figure 7:**
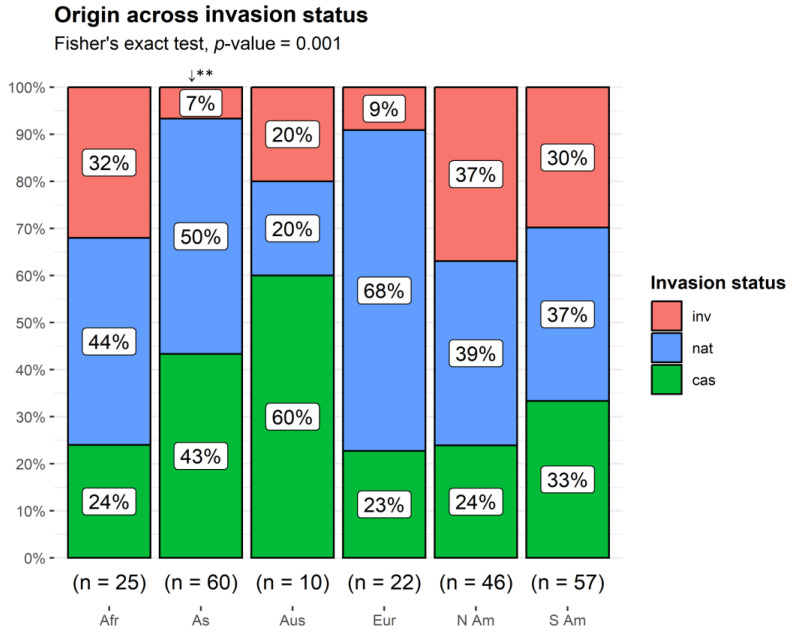
Proportion of non-native taxa by native origin across invasion status categories. Afr: Africa; As: Asia; Aus: Australia; Eur: Europe; N Am: North America: S Am: South America. cas: casual; nat: naturalized; inv: invasive taxa. **↓** observed counts lower than expected by chance. ** *p* < 0.01.

Most of the taxa were of Asian (32%) and S. American (30%) origin, followed by N. American (25%), African (13%), European (12%), and Australian (5%) (Figure 6). The taxa of Asian origin were less frequently observed as invasives than expected by chance (Fisher’s exact test, *p* = 0.001) (Figure 7). South African taxa predominated among the taxa with African origin, covering 40% of them and primarily being invasive (60%). Concerning the European taxa, most of them were specifically of Mediterranean origin (59%) and mainly naturalized, comprising, however, only 7% of the whole alien flora.

The established taxa have their native range predominantly in the Americas (60%), followed by Asian taxa (28%).

### 2.6. Introduction Pathways

A total of 300 introduction pathways were defined for the 187 non-native taxa of Lesvos Island, corresponding on average to 1.6 pathways per taxon (*SD* = 0.65). Most of the taxa (52%) were polyvectic, i.e., utilized more than one introduction pathway. On average, casual, naturalized, and invasive taxa were introduced via analogous numbers of known pathways per taxon (*M* = 1.58, *SD* = 0.68, *M* = 1.62, *SD* = 0.67, and *M* = 1.62, *SD* = 0.58, respectively).

**Figure 8:**
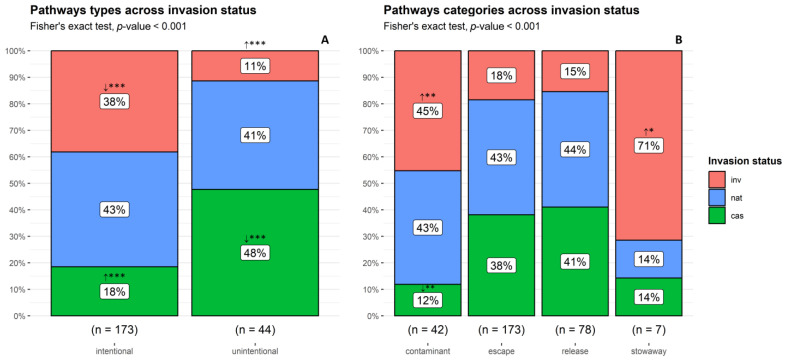
Proportion of non-native taxa by (**A**) pathway types and (**B**) pathway categories across invasion status categories. cas: casual; nat: naturalized; inv: invasive taxa. **↑** observed counts higher than expected by chance; **↓** observed counts lower than expected by chance. *** *p* < 0.001; ** *p* < 0.01; * *p* < 0.05.

Deliberate introductions were responsible for the entry of 93% of the alien plants on Lesvos Island, while 24% were unintentionally introduced. The ratio of unintentional to intentional introductions was lower in archaeophytes, where one out of seven taxa was unintentionally introduced. Regarding invasion status categories, there were significant differences (Fisher’s exact test, *p* < 0.001) in the counts of taxa per pathway type (deliberate vs. unintentional introductions). More casuals than expected by chance were introduced via intentional and fewer via unintentional pathways, while for invasives, the pattern was reversed, as more taxa than expected by chance were introduced via unintentional and fewer via deliberate pathways (Figure 8A).

The escape from confinement pathway was involved in nearly all cases (93%) and in all deliberate introductions, with most of them resulting from ornamental, horticultural, or agricultural plantings (122, 97, and 70 taxa. respectively). The release in nature pathway was responsible for 42% of the deliberate taxa introductions, with most of them concerning landscape/flora “improvement” in the wild. For the unintentional introductions, the transport-contaminant pathway was responsible for 23% of the introductions, with most of them regarding seed contamination, while the transport-stowaway pathway was responsible for only 4% of the taxa, mostly attached to people and their luggage/equipment and machinery/equipment.

The invasion status categories displayed significant differences in the distribution of pathway categories (Fisher’s exact test, *p* < 0.001). Casuals followed the transport-contaminant pathway less often than expected by chance, while invasives introduced as transport contaminants and transport stowaways were more frequent than expected (Figure 8B).

**Figure 9:**
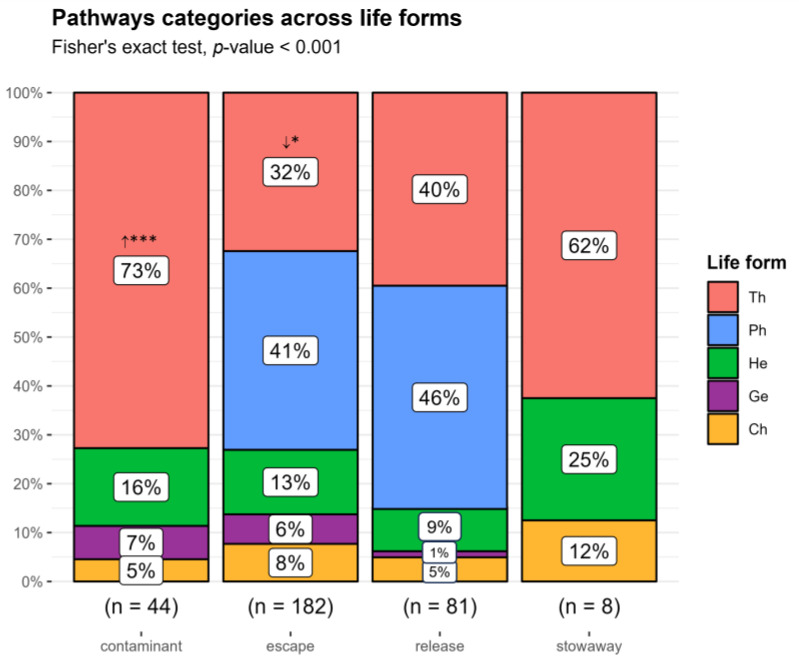
Proportion of non-native taxa by pathway category across life form types. Ch: chamaephyte; Ge: geophyte; He: hemicryptophyte; Ph: phanerophyte; Th: therophyte. **↑** observed counts higher than expected by chance; **↓** observed counts lower than expected by chance. *** *p* < 0.001; * *p* < 0.05.

The distribution of pathway categories displayed significant differences across life forms (Fisher’s exact test, *p* < 0.001) due to therophytes. Therophytes followed the transport-contaminant pathway more often than expected by chance and were less frequent than expected in the escape from confinement (Figure 9).

## 3. Discussion

### 3.1. Species-Area Curves

The present study reports 187 non-native plant taxa (including casuals) which correspond to ca. 12% of the total flora on Lesvos Island. This percentage is comparable with data reported for Stromboli, Salina, Crete, Panarea, the uninhabited island of Cabrera, and Filicudi. It is considerably higher than the corresponding values reported for the Tuscan Archipelago islands, the much smaller and uninhabited island of Dragonera, Rhodes, and the whole of Greece. However, regardless of size, it is lower than other Mediterranean islands, such as Mallorca, Menorca, Linosa, Formentera, Sardinia, Sicily, or Vulcano (Table S2).

The 187 non-native plant taxa of Lesvos Island fall short of the alien species numbers reported for larger Mediterranean islands such as Sicily, Sardinia, Corsica, Crete, or Mallorca; the number is also lower than that on some smaller islands such as Menorca or Ibiza (Table S2). Despite the lack of a mechanistic understanding, it is well known that species richness tends to increase with area, and alien plants appear to conform to this general pattern. In particular, reported SAR intercepts (c-values) are lower for non-native species compared to native species, while SAR exponents (z-values) have been found to be similar between them, though higher across islands compared to the mainland [27]. Conclusively, it seems that alien species richness increases with area at the same rate as native species richness (similar slopes) but accumulates faster on islands than on the mainland (steeper slopes).

**Figure 10:**
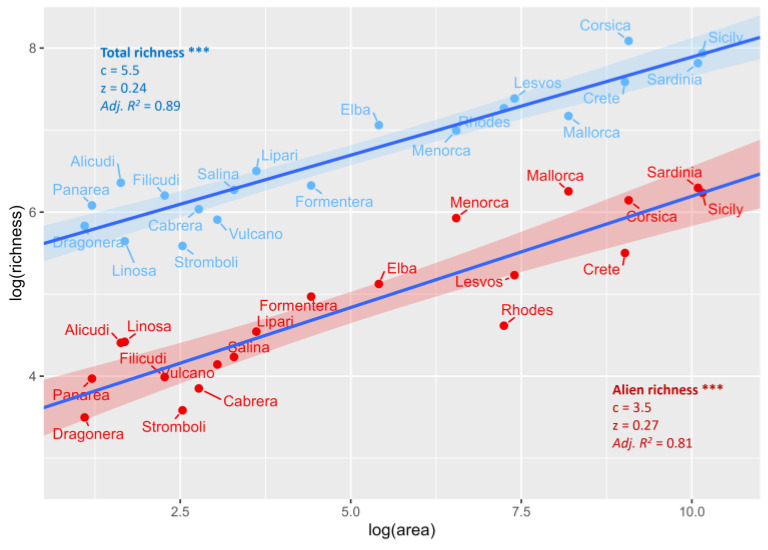
Species–area curves (log–log) of total vs. non-native plant species richness for twenty Mediterranean islands. Shaded areas represent the 95% confidence intervals around the lines. Blue area: total richness; red area: non-native richness. *** *p* < 0.001.

In our study, the species–area curves of total and alien species richness for twenty Mediterranean islands (Table S2, Figure 10) confirmed a lower intercept for non-natives compared to natives (3.5 vs. 5.5), which indicates relatively higher alpha diversity for the native flora on these islands [28]. However, with a z-value = 0.27, within the range of 0.2–0.45 proposed by [29] for insular systems, the slope was steeper for non-native flora. Area explained a greater proportion of the variance in total species richness (Adjusted *R*^2^ = 0.89) compared to that for aliens (Adjusted *R*^2^ = 0.81), which aligns with expectations, likely reflecting the influence of heavily colonized and possibly disturbed anthropogenic zones, along with poorly invaded and possibly less disturbed natural areas [30]. This outcome, besides reflecting an alerting faster accumulation rate for alien species, reveals higher β-diversity for non-native flora among the Mediterranean islands. In other words, it indicates low levels of similarity between islands and a more aggregative distribution pattern [28] as a result of the spatial environmental and anthropogenic mosaics that these Mediterranean islands form.

Still, the number of alien species in an area depends on the combined effect of macroecological, biogeographical, and anthropogenic determinants at local, regional, and global scales. An area can be related to a broad range of the above determinants, such as climate, resource heterogeneity, or habitat diversity. Moreover, it can be linked to human population size and its consequent land use intensity, trade volume, or disturbances that increase propagule pressure in that area [31]. On Lesvos Island, anthropogenic determinants associated with introduction pressure and the establishment potential of non-native plant species have been found to regulate their spatial distribution [16].

### 3.2. Taxonomy

The richest families of Lesvos Island’s alien flora, i.e., Fabaceae, Asteraceae, Poaceae, Amarantaceae, and Solanaceae, are likewise dominant in the alien flora of Greece [32] and also in other alien floras in Europe [33,34], China [35], and globally [36]. Fabaceae, Asteraceae, and Poaceae are also the most abundant families in the native flora of Lesvos, in the Mediterranean Biome [10], and in the Mediterranean basin [37]. These families include many cultivated species and additionally contain species with invasiveness-favoring traits such as a high reproductive rate and grazing resistance (Asteraceae), resilience (Poaceae), or increased dispersal ability (Poaceae and Fabaceae) [10]. In our results, Fabaceae and Poaceae account for the highest number of casual taxa (nine and five, respectively).

Compared to the global naturalized alien flora [36], Lesvos aliens include 17 taxa from the top 50 and 40 taxa from the top 200 most widely distributed naturalized aliens of the world, although in various invasion statuses. Furthermore, of the 30 most widely distributed invasive aliens worldwide, seven taxa (*Lantana camara*, *Leucaena leucocephala*, *Lonicera japonica*, *Eleusine indica*, *Sorghum halepense*, *Arundo donax*, and *Xanthium spinosum*) also occur as aliens on Lesvos; three of them (*L*. *camara*, *L*. *leucocephala*, and *L*. *japonica*) are in the top 10, as naturalized, casual, and naturalized aliens respectively, while *E*. *indica* and *X*. *spinosum* are also considered invasive. Counting the Mediterranean zonobiome alone [36], from the top 10 naturalized species with the highest frequency of occurrence, two taxa (*Erigeron bonariensis* and *Amaranthus hybridus*) also occur on Lesvos as invasives.

Fifteen taxa of the Lesvos alien flora are also included in the National List of Alien Invasive Species (HELLAS-ALIENS) [38], while two taxa (*Acacia saligna* and *Ailanthus altissima*) are included in the third update of the list of Invasive Alien Species (IAS) of Union Concern.

### 3.3. Life Forms

Phanerophytes and therophytes are the prevailing life forms for non-native plants on Lesvos Island. This pattern, common to Mediterranean islands, may reflect two different plant strategies for coping with the stressful typical long dry seasons of the Mediterranean climate: tolerance for phanerophytes and avoidance for therophytes [39]. The biological spectrum of Lesvos Island is quite similar to the one found in the Mediterranean basin, for all life forms but phanerophytes [10], and to those found for Mallorca, and Ibiza, particularly for phanerophytes, hemicryptophytes, and chamaephytes [40].

Non-native phanerophytes are generally associated with direct anthropogenic introductions for forestry, horticulture, and ornamental/gardening purposes [10,40] and are expected to expand their range due to land use changes, both in agricultural and urban environments [41]. Therophytes constitute a high proportion of weeds that are accidentally introduced to ruderal and agricultural habitats. On Lesvos, non-native phanerophytes have been exclusively introduced via deliberate pathways, particularly via escape from confinement, for ornamental (85% of taxa) and horticulture (78% of taxa) purposes, and via release in nature, for landscape/flora/fauna improvement (97% of taxa). Likewise, nearly half of therophytes have been introduced via the transport-contaminant pathway, with most of them (94% of the taxa) as seed contaminants.

Major differences have been observed in the biological spectrum between the non-native and native flora of Lesvos. Aliens display a notably higher proportion of phanerophytes (40% vs. 7%) and lower proportions of therophytes (36% vs. 49%), hemicryptophytes (16% vs. 21%), and geophytes (6% vs. 15%) [42]. The biological spectrum of Lesvos aliens also differs from that of the alien flora of Greece [32], which reveals fewer phanerophytes (22%), and more therophytes (44%) and geophytes (9%).

The proportion of woody alien taxa (48%) is analogically higher than that in the local native flora (14%; [42]) and in the alien flora of Greece (28%; [32]). Naturalized woody taxa (16%) constitute a smaller proportion of the island’s alien flora than that reported for the total and insular global alien taxa (32% and 34%, respectively [36]) and for the alien flora of Greece (32%; [32]).

### 3.4. Density and Naturalization Index

Alien flora density, defined as species per unit area (e.g., taxa/km^2^), can be regarded as a proxy for invasion intensity, reflecting the extent or severity of alien invasion within ecosystems [43]. It is assumed that the higher the density of IAS in an area, the higher their ability to change the structure and diversity of native species communities and thus decrease the function and services of the ecosystem. Alien flora density assumes a constant, proportional relationship between richness and area. This linearity oversimplifies ecological complexity and invasion dynamics, overlooking factors like habitat heterogeneity or disturbance regime history. However, this metric allows for straightforward and standardized comparisons across different-sized regions, highlighting areas under ecological stress and human pressures that should be prioritized for management. Therefore, despite its limitations, this approach, alongside traditional SAR analysis, could be still useful in revealing local invasion pressures. Notably, Lesvos, with a value of 0.11, has one of the lowest alien flora densities among Mediterranean islands and exceeds only the values for Sicily, Sardinia, Crete, Corsica, and Rhodes, while the corresponding density for Greece is very low (Table S2).

The naturalization rate, defined as the proportion of naturalized to all alien taxa (including casuals), for Lesvos is 0.42 or 42%. This is considerably lower than that of Rhodes, Elba, Linosa, Sicily, and Greece as a whole, comparable to Cabrera, Tuscan archipelago islands, Menorca, and Formentera, and larger than that of Dragonera, Crete, Balearics, Ibiza, Mallorca, Sardinia, and Corsica (Table S2). Since naturalized species lay one step in the INIC before becoming invaders, the proportion of naturalized taxa in an alien flora requires special attention for the efficient management of invasive species as it provides a better understanding of invasion processes, species invasiveness, and invasibility at community or regional scale [44]. In addition, introduction pathways should be synchronously considered since fluctuations in naturalization rates among groups of species connected to different aspects of human activities and thus to different pathways have been reported [45].

As invasions are often prompted by stochastic events, residence time is positively connected to invasion success [46]. The longer a species is present in a region, its chances of overcoming various barriers to invasion and shifting its position to the INIC or expanding its range increase, with propagule pressure playing a key role in that [47]. In our data, there is a clear prevalence of neophytes on archaeophytes both for invasive (21% vs. 1%) and naturalized (29% vs. 13%) taxa. Similar trends for invasive neophytes, though in lower proportions, have been reported for Sardinia (12% vs. 1%; [48]) and the Balearics (13% vs. 1%; [49]), while analogous proportions have been shown for Corsica (20% vs. 1%; [48]) and the Tuscan archipelago (20% vs. 1%; [50]).

### 3.5. Reproductive and Dispersal Traits

While pollinator limitation is not considered a significant constraint for the spread of non-native plants, pollination syndrome can be regarded as an invasion stage-dependent trait. A study of Czech Republic flora revealed that in the early stages of invasion, pollination by insects prevails, while in the subsequent stages, wind- and self-pollinated species become more common [51]. A likely interpretation for that shift could be introduction bias, as species with showy flowers, and thus probably insect-pollinated, are frequently preferred for ornamental and horticultural purposes. In our study, more casuals were insect-pollinated (59%) compared to naturalized (57%) and invasive taxa (50%), while garden and horticultural escapes, which would be expected to be most likely insect-pollinated, decreased from 53% among casuals to 51% and 35% among naturalized and invasive taxa, respectively.

It has been suggested that among other factors, the chances of an alien reaching a suitable site depend upon its mobility; thus, successful alien species are usually better dispersers than non-successful ones [52]. Moreover, alien species that can spread rapidly in an area are frequently associated with both natural and anthropogenic dispersal modes [53]. Most taxa in our data relied on natural dispersal strategies, but zoochory, which was the top dispersal mechanism, embraced, in our case, anthropochory as well. Thus, a rapid expansion of range is something we could expect, especially since 52% of invasive and 47% of naturalized taxa used zoochory.

### 3.6. Origin

Most of Lesvos alien plants (55%) originate from the Americas, which is a common pattern for many Mediterranean islands [e.g., Tuscan (43%; [50]), Sardinia (34%; [48]), Corsica (29%; [48]) or Rhodes (21%; [54])], but also for Greece (36%; [32]). Regarding the naturalized taxa only, fewer taxa of American origin were observed on Lesvos compared to the Mediterranean Basin [10] (39% vs. 55%, respectively).

### 3.7. Introduction Pathways

Shrubs and trees exclusively, along with 95% of the perennial taxa, were introduced via intentional pathway categories (escape from confinement and release in nature), exposing their primary use for horticulture or ornamental purposes. In general, direct human involvement in the introduction of aliens is believed to promote their invasiveness, even though unintentional introductions refer to equally widespread species [55]. Our findings, although missing a temporal aspect, do not support this pattern as in our dataset, there were fewer invasive taxa intentionally introduced than expected. Moreover, concerning unintentional introductions, invasive taxa were over-represented among both contaminants and stowaways, which stand out for their superior dispersal ability.

## 4. Materials and Methods

### 4.1. Study Area

**Figure 11:**
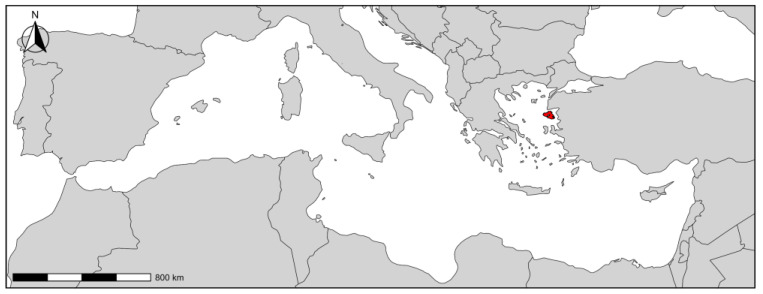
Map of the Mediterranean Basin with Lesvos Island indicated in red. The map was created in RStudio (v.2023.6.0.421) using the *ggplot2* (v.3.4.2) [63] and *sf* (v.1.0.19) [64] packages.

Lesvos Island, located in the NE Aegean Sea, is the 3rd largest island in Greece and the 8th largest in the Mediterranean Basin (Figure 11). With an area of 1636 km^2^ and a coastline of ca. 400 km, it hosts about 90,000 inhabitants. Lesvos is a semi-mountainous island with two high peaks, Lepetymnos (968 m a.s.l.) in the north and Olympos (967 m a.s.l.) in the south, and no major rivers or lakes. The climate is typical Mediterranean (Csa subtype of the Köppen–Geiger classification), with large spatial and seasonal fluctuations due to the regional effect of mountains and atmospheric circulation patterns [56]. The average annual rainfall ranges from 415 mm in the west to 725 mm in the east, while the average annual temperature reaches 17.7 °C. These patterns form three climatic zones: one semi-arid in the west, one dry sub-humid in the central and east, and a transitional semi-arid zone in between them [57]. Lesvos is dominated by volcanic rocks and crystalline schists [58] and used to be joined to the Anatolian mainland until late Palaeolithic/Mesolithic times. It presents an exceptional geological heterogeneity that is reflected in its vegetation [59,60], which includes pine, oak, and chestnut forests, scattered and widespread Mediterranean formations (phrygana and maquis), and important wetlands and riparian zones, as well as extended olive groves and arable lands at lowlands [59,60]. The native flora of Lesvos Island is estimated at 1611 taxa [61]. Recent archaeological excavations on Lesvos revealed traces of human presence since the Paleolithic era [62]. Human activity appears to have been continuous since then, shaping the landscape and vegetation of the island. Over the last decades, there has been a steady decline in the agricultural sector in favor of tourism.

### 4.2. Data Sources and Terminology

This is the first attempt to synthesize the non-native flora of Lesvos Island in order to compile a checklist of Lesvos alien plants. Data were gathered in two ways: (i) by a systematic literature review and (ii) by a thorough roadside survey, a technique that is considered both time- and cost-effective [65,66,67].

The literature review included various sources including peer-reviewed journals (e.g., Phytologia Balcanica, Wildenowia, Bocconea, etc.), dedicated floras and plant checklists (e.g., [68,69]), and the relational database “Alien Plants in Greece: A web-based platform” (https://www.alienplants.gr, accessed on 18 September 2024; [32]). The roadside survey included all main and secondary roads, as well as several mountainous, agricultural, riparian, and coastal footpaths, thus covering most of the island’s surface. Following species’ phenological phases [16], the survey took place in four sampling rounds between 2020 and 2021, while sporadic records were added through 2022. Species presence along with descriptive and spatial data were entered in the open-source QField mobile GIS app (OPENGIS.ch) during the fieldwork [16].

For the compilation of the database, a taxon was included in the checklist if there was any record (bibliographic and/or roadside survey) affirming at least one wild locality of it on Lesvos Island. Moreover, taxa were included when they were commonly cultivated in human-mediated sites such as fields, roadsides, gardens, parks, etc. [70]. Species names were standardized and attributed to families according to World Flora Online (v.2023.03) [71], with records being checked by using the R package *WorldFlora* (v.1.13.2) [72].

For “alien”, we followed the definitions of Pyšek et al., 2004 [46], i.e., plants present in any part of Greece that have arrived with or without human intervention from an area in which they are alien. Translocated species were not considered. Concerning the taxa’s invasion status, i.e., the stage they have reached along the introduction–naturalization–invasion continuum, the taxa were classified into “casual”, “naturalized”, and “invasive” as in [73]. For the residence time status, i.e., information on how long an alien species has been introduced in the region, the taxa were distinguished as “archaeophytes” and “neophytes” (presumably introduced before or after 1492, respectively). Regarding chorology, the taxa were grouped by their geographical origin (native range) at the continent level, including Europe (other than Greece), Africa, Asia, America, and Australia. More precise biogeographical information is discussed, when necessary, in the text. For the analyses of taxa introduction pathways, the classification of the Convention on Biological Diversity [74] was adopted. The specific scheme implements six main categories that are further divided into various subcategories.

Values for plant life-history traits were primarily obtained from [32]. For life forms, we used Raunkiaer’s system classification, which is regarded as the most accepted and utilized system in the world due to its simplicity and efficiency, with the exception of warmer climates such as subtropics or tropics [75]. Residence and invasion status information mainly followed [32] and refers to the whole of Greece. Chorological information was mainly gathered from [32,76], while introduction pathway data were mostly obtained from [77]. Additional sources included the literature, online databases, and field observations.

Some of the taxa were linked with more than one life-history trait, origin, habitat, or pathway category; in such cases, the taxa were considered representative of each category. The Lesvos alien plants checklist was archived on the Global Biodiversity Information Facility (GBIF) [78].

### 4.3. Data Analyses

To test for significant differences in the representation of life-history traits, residence status, origin, habitat preferences, and introduction pathways of the taxa among the invasion status categories, their counts were analyzed by row × column contingency tables (i.e., life form × status) using Fisher’s exact test. To determine for which taxa the counts were lower or higher than expected by chance, adjusted standardized residuals of Chi-square tests were compared with critical values of the normal distribution. All analyses were performed in RStudio (v.2023.6.0.421) [79].

## 5. Conclusions

This publication provides the first comprehensive survey of non-native plants for Lesvos Island in Greece, using a combined method of a literature review and a roadside survey. We anticipate that this work will serve as a valuable reference for comparative studies across Mediterranean islands while paving the way for further investigation of the critical issue of island invasion dynamics. Our results highlight that Lesvos, although hosting several of the most widely distributed naturalized and invasive aliens of the world, compared to other Mediterranean islands, displays a relatively low density of alien species with an intermediate rate of naturalization. Most of the taxa prefer disturbed ruderal and agricultural habitats, whereas the frequently prone to invasion freshwater and coastal habitats exhibit low numbers of aliens. Deliberate introduction pathways are the major source of alien species, with unintentional introductions favoring particular invasive taxa.

As land abandonment and tourism development increase, more alien species are likely to enter the island. Furthermore, already present non-native taxa are expected to establish or increase their spread and consequential impacts on natural ecosystems, human or other animal health, and the economy. Although still less invaded, Mediterranean islands are more prone to invasions than analogous mainland areas. This is critical for conservation efforts and indicates the importance of such studies and the need for a comprehensive management strategy that will include monitoring, prevention, early detection, and control actions towards non-native invasive species.

## Data Availability

The original data presented in this study are available in GBIF—the Global Biodiversity Information Facility—at https://doi.org/10.15468/wj8syb.

## Supplementary Materials

The following supporting information can be downloaded at https://www.mdpi.com/article/10.3390/plants13233375/s1: Table S1: Alien taxa checklist of Lesvos Island with information on record type; Table S2: Area, total and non-native plant species richness, and naturalized non-native taxa for twenty-three Mediterranean islands and Greece; Table S3: Contingency tables of the counts of taxa per categorical trait between invasion status categories. Refs. [80,81,82,83,84] are cited in the Supplementary Materials.
