# Supplementary material for: Non-Native Flora of the Mediterranean Lesvos Island (East Aegean, Greece): Floristic Analysis, Traits, and Assessment
Source: Plants (Basel). 2024 Nov 30;13(23):3375. doi: 10.3390/plants13233375 (PMC11644495; doi:10.3390/plants13233375)
Supplement: Supplementary file 1 [file plants-13-03375-s001.zip › plants-3274843-supplementary.pdf]

Table S1: *Alien taxa checklist of Lesvos Island with information on record type.* [1]*: new record for Lesvos;* [2]*: new record for Greece.*

Family	*Taxon*	*Record type*
Acanthaceae	*Acanthus mollis* L.	field
Acanthaceae	*Justicia adhatoda* L.	field[1]
Aizoaceae	*Carpobrotus edulis* (L.) N.E.Br.	field
Aizoaceae	*Malephora purpureocrocea* (Haw.) Schwantes	field[1]
Aizoaceae	*Mesembryanthemum cordifolium* L.f.	field
Amaranthaceae	*Amaranthus albus* L.	field
Amaranthaceae	*Amaranthus blitoides* S.Watson	field
Amaranthaceae	*Amaranthus blitum* subsp. *emarginatus* (Salzm. ex Uline & W.L.Bray)	literature
Amaranthaceae	*Amaranthus caudatus* L.	literature
Amaranthaceae	*Amaranthus cruentus* L.	literature
Amaranthaceae	*Amaranthus deflexus* L.	field
Amaranthaceae	*Amaranthus hybridus* subsp. *quitensis* (Kunth) Costea & Carretero	literature
Amaranthaceae	*Amaranthus hypochondriacus* L.	literature
Amaranthaceae	*Amaranthus powellii* S.Watson	literature
Amaranthaceae	*Amaranthus powellii* subsp. *bouchonii* (Thell.) Costea & Carretero	literature
Amaranthaceae	*Amaranthus quitensis* Kunth	literature
Amaranthaceae	*Amaranthus retroflexus* L.	field
Amaranthaceae	*Amaranthus viridis* L.	literature
Amaryllidaceae	*Narcissus tazetta* subsp. *aureus* (Loisel.) Baker	literature
Amaryllidaceae	*Nothoscordum gracile* (Aiton) Stearn	literature
Anacardiaceae	*Pistacia vera* L.	literature
Anacardiaceae	*Schinus molle* L.	field
Apiaceae	*Anethum graveolens* L.	field
Apiaceae	*Pimpinella anisum* L.	field[1]
Apocynaceae	*Catharanthus roseus* (L.) G.Don	field
Apocynaceae	*Gomphocarpus fruticosus* (L.) W.T.Aiton	literature
Apocynaceae	*Gomphocarpus physocarpus* E.Mey.	literature
Arecaceae	*Phoenix dactylifera* L.	field
Asparagaceae	*Agave americana* L.	field
Asparagaceae	*Agave americana* L. subsp. *americana*	field[1]
Asparagaceae	*Hyacinthus orientalis* L.	literature
Asparagaceae	*Yucca gloriosa* L.	field
Asphodelaceae	*Aloe vera* (L.) Burm.f.	field[1]
Asteraceae	*Bidens bipinnata* L.	literature
Asteraceae	*Calendula officinalis* L.	field
Asteraceae	*Cotula coronopifolia* L.	literature
Asteraceae	*Cynara cardunculus* L.	field[1]
Asteraceae	*Erigeron bonariensis* L.	field
Asteraceae	*Erigeron canadensis* L.	field
Asteraceae	*Erigeron sumatrensis* Retz.	field
Asteraceae	*Helianthus annuus* L.	field
Asteraceae	*Helianthus laetiflorus* Pers.	literature
Asteraceae	*Symphyotrichum novi-belgii* (L.) G.L.Nesom	literature

Asteraceae	*Symphyotrichum subulatum* var. *squamatum* (Spreng.) S.D.Sundb.	literature
Asteraceae	*Tagetes minuta* L.	literature
Asteraceae	*Tagetes erecta* L.	literature
Asteraceae	*Xanthium orientale* subsp. *italicum* (Moretti) Greuter	field
Asteraceae	*Xanthium spinosum* L.	field
Bignoniaceae	*Campsis radicans* (L.) Seem.	field
Bignoniaceae	*Catalpa bignonioides* Walter	field[1]
Bignoniaceae	*Dolichandra unguis-cati* (L.) L.G.Lohmann	field[1]
Bignoniaceae	*Jacaranda mimosifolia* D.Don	field[1]
Bignoniaceae	*Podranea ricasoliana* (Tanfani) Sprague	field[1]
Boraginaceae	*Heliotropium curassavicum* L.	field
Brassicaceae	*Brassica rapa* subsp. *chinensis* (L.) P.Hanelt	literature
Brassicaceae	*Erysimum cheiri* (L.) Crantz	literature
Brassicaceae	*Lepidium didymum* L.	literature
Buddlejaceae	*Buddleja asiatica* Lour.	field
Buddlejaceae	*Buddleja davidii* Franch.	field
Cactaceae	*Austrocylindropuntia subulata* (Muehlenpf.) Backeb.	field[1]
Cactaceae	*Cereus jamacaru* DC.	field[2]
Cactaceae	*Opuntia ficus-indica* (L.) Mill.	field[1]
Cactaceae	*Opuntia microdasys* (Lehm.) Pfeiff.	field[1]
Cactaceae	*Opuntia monacantha* Haw.	field[1]
Cactaceae	*Opuntia robusta* H.L. Wendl. ex Pfeiff.	field
Cannabaceae	*Cannabis sativa* L.	literature
Caprifoliaceae	*Lonicera japonica* Thunb.	field
Casuarinaceae	*Casuarina equisetifolia* L.	field
Chenopodiaceae	*Bassia scoparia* (L.) A.J.Scott	literature
Chenopodiaceae	*Chenopodium giganteum* D.Don	literature
Chenopodiaceae	*Dysphania ambrosioides* (L.) Mosyakin & Clemants	literature
Chenopodiaceae	*Dysphania multifida* (L.) Mosyakin & Clemants	literature
Commelinaceae	*Commelina communis* L.	literature
Convolvulaceae	*Cuscuta campestris* Yunck.	field
Convolvulaceae	*Dichondra micrantha* Urban	literature
Convolvulaceae	*Ipomoea hederacea* Jacq.	field
Convolvulaceae	*Ipomoea indica* (Burm.) Merr.	field
Convolvulaceae	*Ipomoea purpurea* (L.) Roth	field
Cucurbitaceae	*Citrullus lanatus* (Thunb.) Matsum. & Nakai	field
Cucurbitaceae	*Cucumis melo* L.	field[1]
Cucurbitaceae	*Cucurbita maxima* Lam.	field[1]
Cucurbitaceae	*Cucurbita pepo* L.	field
Cyperaceae	*Cyperus involucratus* Rottb.	field[1]
Elaeagnaceae	*Elaeagnus angustifolia* L.	field
Euphorbiaceae	*Euphorbia maculata* L.	literature
Euphorbiaceae	*Euphorbia prostrata* Aiton	literature
Euphorbiaceae	*Ricinus communis* L.	field
Fabaceae	*Acacia dealbata* Link	field
Fabaceae	*Acacia farnesiana* (L.) Willd.	field[1]

Fabaceae	*Acacia saligna* (Labill.) H.L.Wendl.	field
Fabaceae	*Amorpha fruticosa* L.	literature
Fabaceae	*Cicer arietinum* L.	literature
Fabaceae	*Erythrostemon gilliesii* (Hook.) Klotzsch	field[1]
Fabaceae	*Lathyrus sativus* L.	field[1]
Fabaceae	*Lens culinaris* Medik.	field
Fabaceae	*Leucaena leucocephala* (Lam.) de Wit	field
Fabaceae	*Lupinus gredensis* Gand.	literature
Fabaceae	*Parkinsonia aculeata* L.	literature
Fabaceae	*Phaseolus vulgaris* L.	field
Fabaceae	*Pisum sativum* L. subsp. *sativum*	literature
Fabaceae	*Robinia pseudoacacia* L.	field
Fabaceae	*Trigonella caerulea* (L.) Ser.	field[1]
Fabaceae	*Vicia faba* L.	literature
Fabaceae	*Wisteria sinensis* (Sims) Sweet	field
Hydrangeaceae	*Philadelphus coronarius* L.	literature
Iridaceae	*Freesia leichtlinii* Klatt	field
Iridaceae	*Iris florentina* L.	field
Lamiaceae	*Ocimum basilicum* L.	field
Malvaceae	*Alcea rosea* L.	field
Malvaceae	*Brachychiton populneus* (Schott & Endl.) R.Br	field[1]
Martyniaceae	*Ibicella lutea* (Lindl.) Van Eselt.	literature
Meliaceae	*Melia azedarach* L.	field
Mimosaceae	*Albizia julibrissin* Durazz.	field[1]
Moraceae	*Broussonetia papyrifera* (L.) Vent.	field
Moraceae	*Morus alba* L.	field
Moraceae	*Morus nigra* L.	field
Myrtaceae	*Eucalyptus camaldulensis* Dehnh.	field
Nyctaginaceae	*Mirabilis jalapa* L.	field
Oleaceae	*Fraxinus angustifolia* subsp. *syriaca* (Boiss.) Yalt.	field[1]
Oleaceae	*Jasminum officinale* L.	field
Onagraceae	*Clarkia amoena* (Lehm.) A.Nelson & J.F.Macbr.	literature
Onagraceae	*Oenothera lindheimeri* (Engelm. & A.Gray) W.L.Wagner & Hoch	literature
Onagraceae	*Oenothera speciosa* Nutt.	literature
Oxalidaceae	*Oxalis articulata* Savigny	field
Oxalidaceae	*Oxalis articulata* f. *crassipes* (Urb.) Lourteig	field[2]
Oxalidaceae	*Oxalis debilis* Kunth	field
Oxalidaceae	*Oxalis dillenii* Jacq.	literature
Oxalidaceae	*Oxalis pes-caprae* L.	field
Passifloraceae	*Passiflora caerulea* L.	field[1]
Paulowniaceae	*Paulownia tomentosa* (Thunb.) Steud.	field
Phytolaccaceae	*Phytolacca americana* L.	field
Pinaceae	*Pinus pinaster* Aiton	field[1]
Plantaginaceae	*Antirrhinum majus* L.	field
Plantaginaceae	*Antirrhinum majus* L. subsp. *majus*	field
Plantaginaceae	*Antirrhinum majus* subsp. *tortuosum* (Vent.) Rouy	field[1]

Plantaginaceae	*Cymbalaria muralis* G.Gaertn., B.Mey. & Scherb.	field
Plantaginaceae	*Cymbalaria muralis* G.Gaertn., B.Mey. & Scherb. subsp. *muralis*	literature
Plantaginaceae	*Veronica persica* Poir.	literature
Plumbaginaceae	*Plumbago auriculata* Lam.	literature
Poaceae	*Arundo donax* L.	field
Poaceae	*Avena sativa* L.	literature
Poaceae	*Coix lacryma-jobi* L.	field[1]
Poaceae	*Cortaderia selloana* (Schult. & Schult.f.) Asch. & Graebn.	field[1]
Poaceae	*Digitaria ciliaris* (Retz.) Koeler	literature
Poaceae	*Echinochloa colona* (L.) Link	literature
Poaceae	*Eleusine indica* (L.) Gaertn.	literature
Poaceae	*Panicum miliaceum* L.	literature
Poaceae	*Paspalum distichum* L.	literature
Poaceae	*Phalaris canariensis* L.	literature
Poaceae	*Sorghum bicolor* (L.) Moench	literature
Poaceae	*Sorghum halepense* (L.) Pers.	literature
Poaceae	*Triticum aestivum* L.	literature
Poaceae	*Zea mays* L.	field
Portulacaceae	*Portulaca grandiflora* Hook.	literature
Proteaceae	*Grevillea robusta* A.Cunn. ex R.Br.	field[2]
Punicaceae	*Punica granatum* L.	field
Rhamnaceae	*Ziziphus jujuba* Mill.	field
Rosaceae	*Cydonia oblonga* Mill.	field
Rosaceae	*Eriobotrya japonica* (Thunb.) Lindl.	field[1]
Rosaceae	*Malus domestica* Borkh.	field
Rosaceae	*Mespilus germanica* L.	literature
Rosaceae	*Prunus armeniaca* L.	field
Rosaceae	*Prunus cerasus* L.	field
Rosaceae	*Prunus dulcis* (Mill.) D.A.Webb	field
Rosaceae	*Prunus persica* (L.) Batsch	field
Salicaceae	*Populus nigra* L.	literature
Sapindaceae	*Acer negundo* L.	field
Sapindaceae	*Cardiospermum halicacabum* L.	field
Sapindaceae	*Koelreuteria paniculata* Laxm.	field[1]
Simaroubaceae	*Ailanthus altissima* (Mill.) Swingle	field
Solanaceae	*Brugmansia suaveolens* (Willd.) Bercht. & C.Presl	field[1]
Solanaceae	*Datura innoxia* Mill.	field
Solanaceae	*Datura stramonium* L.	field
Solanaceae	*Lycium barbarum* L.	literature
Solanaceae	*Lycium chinense* Mill.	field
Solanaceae	*Nicandra physalodes* (L.) Gaertn.	literature
Solanaceae	*Nicotiana glauca* R.C.Graham	field
Solanaceae	*Solanum elaeagnifolium* Cav.	field
Solanaceae	*Solanum lycopersicum* L.	literature
Solanaceae	*Solanum pseudocapsicum* L.	field
Solanaceae	*Solanum rostratum* Dunal	literature

Solanaceae	*Solanum tuberosum* L.	literature
Tropaeolaceae	*Tropaeolum majus* L.	field[1]
Valerianaceae	*Valeriana macrosiphon* (Boiss.) Bailly	field[1]
Verbenaceae	*Lantana camara* L.	field
Vitaceae	*Parthenocissus quinquefolia* (L.) Planch.	literature
Vitaceae	*Vitis vinifera* L. subsp. *vinifera*	literature

Table S2: *Area, total and non-native plant species richness, naturalized non-native taxa, alien flora density, and naturalization rate for twenty-three Mediterranean islands, and Greece. NA's indicate missing values.*

**Island**	**Area (km²)**	**Total species richness**	**Non-native species richness**	**Naturalized taxa**	**Alien flora density**	**Naturalization rate (%)**	**Data source**
Sicily	25,711	2,801	510	236	0.020	46	[1,2]
Sardinia	24,090	2,479	542	147	0.022	27	[1,2]
Corsica	8,722	3,252	466	117	0.053	25	[3]
Crete	8,259	1,972 [4]	245	91	0.030	37	[5]
Balearics	4,992	1,975	624	219	0.125	35	[6]
Mallorca	3,610	1,302	520	175	0.144	34	[6]
Lesvos	1,636	1,611 [4]	187	79	0.114	42	Current study
Rhodes	1,401	1,431 [4]	101	78	0.072	77	[7]
Menorca	696	1,090	375	155	0.539	41	[6]
Ibiza	541		235	82	0.434	35	[6]
Tuscan	293	1,400	141	62	0.481	44	[8]
Elba	224	1,165	168	101	0.750	60	[9]
Formentera	83	558	144	58	1.735	40	[6]
Lipari	37.2	666	94	NA	2.527	NA	[10]
Salina	26.8	529	69	NA	2.575	NA	[10]
Vulcano	21	368	63	NA	3.000	NA	[10]
Cabrera	16	418	47	21	2.938	45	[6]
Stromboli	12.6	267	36	NA	2.857	NA	[10]
Filicudi	9.7	494	54	NA	5.567	NA	[10]
Linosa	5.4	283	83	49	15.370	59	[11]

Alicudi	5.1	577	82	NA	16.078	NA	[10]
Panarea	3.34	438	53	NA	15.868	NA	[10]
Dragonera	3	341	33	13	11.000	39	[6]
Greece	13,1957	6,846	457	282	0.003	62	[12,13]

1. Galasso, G.; Conti, F.; Peruzzi, L.; Alessandrini, A.; Ardenghi, N.M.G.; Bacchetta, G.; Banfi, E.; Barberis, G.; Bernardo, L.; Bouvet, D.; et al. A Second Update to the Checklist of the Vascular Flora Alien to Italy. *Plant Biosyst. - Int. J. Dea*L. *Asp. Plant Bio*L. **2024**, *158*, 297–340, doi:10.1080/11263504.2024.2320129.
2. Bartolucci, F.; Peruzzi, L.; Galasso, G.; Alessandrini, A.; Ardenghi, N.M.G.; Bacchetta, G.; Banfi, E.; Barberis, G.; Bernardo, L.; Bouvet, D.; et al. A Second Update to the Checklist of the Vascular Flora Native to Italy. *Plant Biosyst. - Int. J. Dea*L. *Asp. Plant Bio*L. **2024**, *158*, 219–296, doi:10.1080/11263504.2024.2320126.
3. Puddu, S.; Podda, L.; Mayoral, O.; Delage, A.; Hugot, L.; Petit, Y.; Bacchetta, G. Comparative Analysis of the Alien Vascular Flora of Sardinia and Corsica. *Not. Bot. Horti Agrobot. Cluj-Napoca* **2016**, *44*, 337–346, doi:10.15835/nbha44210491.
4. Strid, A. The Botanical Exploration of Greece. *Plant Syst. Evo*L. **2020**, *306*, 27, doi:10.1007/s00606-020-01637-z.
5. Dal Cin D'Agata, C.; Skoula, M.; Brundu, G. A Preliminary Inventory of the Alien Flora of Crete (Greece). *Bocconea* **2009**, *23*, 301–315.
6. Cerrato, M.D.; Cortés-Fernández, I.; Ribas-Serra, A.; Mir-Rosselló, P.M.; Cardona, C.; Gil, L. Time Pattern Variation of Alien Plant Introductions in an Insular Biodiversity Hotspot: The Balearic Islands as a Case Study for the Mediterranean Region. *Biodivers. Conserv.* **2023**, *32*, 2585–2605, doi:10.1007/s10531-023-02620-z.
7. Galanos, C.J. The Alien Flora of Terrestrial and Marine Ecosystems of Rodos Island (SE Aegean), Greece. *Willdenowia* **2015**, *45*, 261–278, doi:10.3372/wi.45.45211.
8. Lazzaro, L.; Ferretti, G.; Giuliani, C.; Foggi, B. A Checklist of the Alien Flora of the Tuscan Archipelago (Italy). *Webbia* **2014**, *69*, 157–176, doi:10.1080/00837792.2014.907981.
9. Carta, A.; Forbicioni, L.; Frangini, G.; Pierini, B.; Peruzzi, L. An Updated Inventory of the Vascular Flora of Elba Island (Tuscan Archipelago, Italy). *Ita*L. *Bot.* **2018**, *6*, 1–22, doi:10.3897/italianbotanist.6.26568.
10. Chiarucci, A.; Guarino, R.; Pasta, S.; Rosa, A.L.; Cascio, P.L.; Médail, F.; Pavon, D.; Fernández-Palacios, J.M.; Zannini, P. Species–Area Relationship and Small-Island Effect of Vascular Plant Diversity in a Young Volcanic Archipelago. *J. Biogeogr.* **2021**, *48*, 2919–2931, doi:10.1111/jbi.14253.
11. Pasta, S.; Ardenghi, N.M.G.; Badalamenti, E.; Mantia, T.L.; Console, S.L.; Parolo, G. The Alien Vascular Flora of Linosa (Pelagie Islands, Strait of Sicily): Update and Management Proposals. *Willdenowia* **2017**, *47*, 135–144, doi:10.3372/wi.47.47205.
12. Dimopoulos, P.; Bazos, I.; Kokkoris, I.P.; Zografidis, A.; Karadimou, E.; Kallimanis, A.S.; Raus, T.; Strid, A. *A Guide to the Alien Plants of Greece with Reference to the Natura 2000 Protected Area Network*; Natural Environment and Climate Change Agency (NECCA): Athens, 2020; ISBN 978-618- 85104-1-8.
13. Dimopoulos, P.; Raus, T.; Bergmeier, E.; Constantinidis, T.; Iatrou, G.; Kokkini, S.; Strid, A.; Tzanoudakis, D. *Vascular Plants of Greece: An Annotated Checklist*; Englera; Botanic Garden And Botanical Museum Berlin-dahlem; Hellenic Botanical Society: Berlin; Athens, 2013; ISBN 978-3-921800-88-1.

Table S3: *Contingency tables of the counts of taxa per categorical trait between invasion status categories (Table S3a-h) and life forms (Table S3i).*

Table S3a: *Residence status. arch: archaeophytes; neo: neophytes. cas: casual; nat: naturalized; inv: invasive taxa.*

Residence	*inv*	*nat*	*cas*	*Sum*
arch	2	24	9	35
neo	40	55	57	152
Sum	42	79	66	187
	Fisher's Exact Test p-value < 0.001			

Table S3b: *Growth form. H: herbs; S: shrubs; SS: subshrubs; T: trees. cas: casual; nat: naturalized; inv: invasive taxa.*

Growth form	*inv*	*nat*	*cas*	*Sum*
H	31	50	27	108
S	5	15	20	40
SS	5	5	1	11
T	4	12	22	38
Sum	45	82	70	197
	Fisher's Exact Test p-value < 0.001			

Table S3c: *Life form. Ch: chamaephytes; Ge: geophytes; He: hemicryptophytes; Ph: phanerophytes; Th: therophytes. cas: casual; nat: naturalized; inv: invasive taxa.*

Life form	*inv*	*nat*	*cas*	*Sum*
Ch	7	7	2	16
Ge	3	8	1	12
He	8	14	7	29
Ph	12	23	39	74
Th	18	29	20	67
Sum	48	81	69	198
	Fisher's Exact Test p-value = 0.005			

Table S3d: *Fruit type. Ach: Achene, Ber: Berry, Cap: Capsule, Car: Caryopsis, Con: Cone, Dru: Drupe, Fol: Follicle, Leg: Legume, Sam: Samara, Sch: Schizocarp, Sil: Silicle, Utr: Utricle. cas: casual; nat: naturalized; inv: invasive taxa.*

Fruit type	*inv*	*nat*	*cas*	*Sum*
Ach	11	18	3	32
Ber	5	9	18	32
Cap	26	34	21	81
Car	2	7	5	14
Con	0	0	1	1
Dru	0	7	5	12
Fol	1	1	3	5
Leg	2	6	12	20
Sam	1	2	2	5
Sch	1	2	1	4

Sil	0	1	4	5
Utr	1	0	0	1
Sum	50	87	75	212
	Fisher's Exact Test p-value = 0.001			

Table S3e: *Habitat preferences. A: freshwater; C: cliffs, rocks, walls, ravines, boulders; M: coastal; P: xeric Mediterranean phrygana and grasslands; R: ruderal and agricultural; W: woodlands and scrub. cas: casual; nat: naturalized; inv: invasive taxa.*

Habitat	*inv*	*nat*	*cas*	*Sum*
A	4	5	0	9
C	1	8	1	10
M	5	0	1	6
P	1	1	0	2
R	36	74	66	176
W	1	4	0	5
Sum	48	92	68	208
	Fisher's Exact Test p-value < 0.001			

Table S3f: *Origin. Afr: Africa; As: Asia; Aus: Australia; Eur: Europe; N Am: North America: S Am: South America. cas: casual; nat: naturalized; inv: invasive taxa.*

Origin	*inv*	*nat*	*cas*	*Sum*
Afr	8	11	6	25
As	4	30	26	60
Aus	2	2	6	10
Eur	2	15	5	22
N Am	17	18	11	46
S Am	17	21	19	57
Sum	50	97	73	220
	Fisher's Exact Test p-value = 0.001			

Table S3g: *Introduction pathway types. cas: casual; nat: naturalized; inv: invasive taxa.*

Path type	*inv*	*nat*	*cas*	*Sum*
intentional	32	75	66	173
unintentional	21	18	5	44
Sum	53	93	71	217
	Fisher's Exact Test p-value < 0.001			

Table S3h: *Introduction pathway categories. cas: casual; nat: naturalized; inv: invasive taxa.*

Path category	*inv*	*nat*	*cas*	*Sum*
contaminant	19	18	5	42
escape	32	75	66	173
release	12	34	32	78
stowaway	5	1	1	7
Sum	68	128	104	300
	Fisher's Exact Test p-value < 0.001			

Table S3i: *Introduction pathway categories across Life forms. Ch: chamaephytes; Ge: geophytes; He: hemicryptophytes; Ph: phanerophytes; Th: therophytes. cas: casual; nat: naturalized; inv: invasive taxa.*

Path category	*Ch*	*Ge*	*He*	*Ph*	*Th*	*Sum*
contaminant	2	3	7	0	32	44
escape	14	11	24	74	59	182
release	4	1	7	37	32	81
stowaway	1	0	2	0	5	8
Sum	21	15	40	111	128	315
	Fisher's Exact Test p-value < 0.001					
